# PAI-1 Level Differences in Malignant Plural Effusion, Parapneumonic Pleuritis, and Cardiac Hydrothorax

**DOI:** 10.3390/medicina55090567

**Published:** 2019-09-04

**Authors:** Dace Zentina, Inga Stukena, Alvils Krams, Aivars Lejnieks

**Affiliations:** 1 Department of Internal Diseases, Pauls Stradins University Hospital, Pilsonu Street 13, LV 1002 Riga, Latvia; 2 Department of Internal Diseases, Riga Stradins University, Dzirciema Street 16, LV 1007 Riga, Latvia; 3 Department of Internal Diseases, Riga East University Hospital, Hipokrata Street 2, LV 1038 Riga, Latvia; inga.stukena@gmail.com (I.S.); lejnieks@latnet.lv (A.L.); 4 Centre of Tuberculosis and Lung Disease, Riga East University Hospital, Upeslejas, LV 2118 Stopini region, Latvia; alvils.krams@aslimnica.lv; 5 Department of Internal Disease, Faculty of Medicine, University of Latvia, Jelgavas Street 3, LV 1004 Riga, Latvia

**Keywords:** pleural effusion, malignant pleural effusion, plasminogen activator inhibitor-1

## Abstract

*Background and Objectives:* Plasminogen activator inhibitor-1 (PAI-1) is a fibrinolytic system enzyme whose role in various fibrinolytic processes is currently unknown. In clinical manifestations of pleural liquids of diverse etiology, various levels of fibrinolytic activity can be observed—parapneumonic processes tend to loculate in fibrin septa, while malignant pleural effusion (MPE) does not. The purpose of this study was to determine possible differences in PAI-1 levels in pleural effusions of varied etiology. *Material and Methods:* PAI-1 level in pleural effusion and serum was determined in 144 patients with pleural effusions of various etiology (cardiac hydrothorax—42 patients (29.2%), MPE—67 patients (46.5%), parapneumonic pleuritis—27 (18.8%), tuberculous pleuritis—6 patients (4.1%), pancreatogenic pleuritis—1 patient (0.7%) and pulmonary artery thromboembolism with pleuritis—1 patient (0.7%)). *Results:* The median PAI-1 level (ng/mL) was the highest in the parapneumonic pleuritis group both in the effusion and the serum, with values of 291 (213–499) ng/mL and 204 (151–412) ng/mL, respectively, resulting in a statistically significant difference (*p* < 0.001) from the cardiac hydrothorax and MPE groups. However, there was no statistically significant difference between PAI-1 levels in the pleural effusion and serum in the cardiac hydrothorax and MPE groups. *Conclusion:* The PAI-1 level in MPE and cardiac hydrothorax was statistically significantly lower than in parapneumonic pleuritis.

## 1. Introduction

Plasminogen activator inhibitor-1 (PAI-1) is a specific urokinase type (uPA) and tissue type (tPA) inhibitor of the plasminogen activator.

Both uPA and tPA catalyze the conversion of inactive plasminogen into the active serin protease plasmin, which in turn catalyzes the degradation of the extracellular proteins fibrin and laminin. Consequently, the physiological functions of plasminogen activation include fibrinolysis and the remodeling of tissues. However, it has recently been determined that PAI-1 is involved in many molecular processes that are not directly related to proteolysis [1,2].

Plasmin catalyzes degradation of the basal membrane and extracellular matrix, potentially easing the invasion of cancer cells into the surrounding healthy tissue [3]; thus, it could be expected that the protease inhibitor will limit cancerous growth, invasion, and metastases. However, currently, it is known that a high level of PAI-1 in primary tumors is one of the most accurate biochemical markers for an unfavorable prognosis, especially in breast cancer patients. Therefore, protease inhibitors do not have the expected anti-invasive effect, and even promote the growth of tumors [4].

It is still not clear what role PAI-1 has in the development of tumors. There is a hypothesis that a high level of PAI-1 could protect tumor cells from destruction due to increased proteolysis of normal tissue [1]. Another hypothesis is that PAI-1 participates in neoangiogenesis by binding with the protein vitronectin, and thus inhibits apoptosis [5]. It is possible that various concentrations of PAI-1 have a different effect [1]; thus, it could be expected that in inflammatory and malignant effusions and transudates, there would be various PAI-1 levels.

Considering the above, the purpose of the study was to establish differences in PAI-1 levels in malignant pleural effusion, parapneumonic pleuritis, and cardiac hydrothorax.

## 2. Materials and Methods

A total of 144 patients, who were consecutively admitted to the Pulmonology Department of the Riga East University Hospital Internal Diseases Clinic from 8 August 2011 to 13 June 2014, were enrolled in the study.

Inclusion criteria:

- Pleural effusion confirmed under X-ray or ultrasound control;
- Diagnostic and/or therapeutic indications for thoracentesis;
- Signed informed patient consent.

The following were considered indications for thoracentesis:

1. Pleural effusion of an unclear origin, including suspected MPE;
2. Parapneumonic pleuritis, including suspected empyema;
3. Large amounts of pleural effusion to reduce shortness of breath.

The following were not included in the study:

- Patients repeatedly admitted with pleural effusion;
- Patients with clinical decompensated heart failure (confirmed heart disease in anamnesis, progressing shortness of breath, peripheral edemas, signs of heart failure in an echocardiogram (echoCG), congestion in the pulmonary circuit X-ray) and cardiac hydrothorax, which had no diagnostic or therapeutic indications for thoracentesis.

Thoracentesis was performed following the patient’s signed informed consent, under local anesthesia with *Sol. Lidocaini* 2% and by means of *Pleurocan* (*Braun*) pleural catheters, concurrently performing venipuncture of a peripheral vein. PAI-1 in serum and pleural effusion was determined for all patients by means of cytometric xMAP technology (Luminex device).

In addition, routine tests were performed for the patients, including lactate dehydrogenase (LDH) and proteins in pleural effusion and serum and cytological analyses of the pleural effusion.

Having assessed the Light’s criteria, the patients were divided into two groups according to type of pathogenesis of the pleural effusion: transudates and exudates. Patients with signs of heart failure were included in the cardiac hydrothorax (transudate) group. Patients with parapneumonic pleuritis were diagnosed according to the following criteria: acute disease with cough, a febrile temperature, raised levels of inflammatory markers (CRO), and respective X-ray findings. Malignant pleural effusion (MPE) was diagnosed on the basis of cytological analyses of the pleural effusion. If cytological analyses were negative, the effusion was only believed to be MPE in cases when the patient already had a confirmed malignant disease and no other cause for the effusion was found [6]. Patients whose number of monomorphic nuclear cells in the clinical analyses of the pleural effusion was >95% had a closed pleural biopsy. Diagnosis of tuberculous pleuritis was initially established by means of a histological analysis of pleural biopsy by finding specific changes—epithelioid cell granulomas with caseous necrosis, confirming the above with a bacteriological analysis of the bioptate. Pancreatitis-related pleuritis was diagnosed on the basis of an elevated level of lipase in the pleural effusion.

All statistical calculations were made with SPSS (Statistical Package for the Social Sciences) for Windows, Version 23.0, and MS Excel 2007. According to the general principles of medical statistics, a *p*-value of 0.05 was considered the threshold of statistical reliability for the results of bilateral tests. General statistical methods were used to describe groups of subjects. The distribution of quantitative data was checked using histograms and the Kolmogorov–Smirnov test to establish whether or not it was normal. As the distribution of data was not normal, the median, 25th, and 75th percentiles were used to describe the average indicators.

### Ethical Approval

The study was allowed by the Ethics Committee of Riga Stradins University (date of approval 23 September 2010, number: E-9 (2)).

## 3. Results

From 8 August 2011 to 13 June 2014, 144 patients, who were consecutively admitted to the Pulmonology Department of the Internal Diseases Clinic Riga East University Hospital, complied with the inclusion criteria. A total of 69 (47.9%) of them were males. The patients were 22 to 97 years of age.

It was established that according to the etiology of the pleural effusion, the examined patients could be grouped as follows: in the cardiac hydrothorax (all patients had transudate according to Light’s criteria) group—42 patients (29.2%), 22 (52.3%) of them males; in the MPE group—67 patients (46.5%), 21 (31.3%) of them males; in the parapneumonic pleuritis group—27 patients (18.8%), 19 (70.3%) of them males. In six patients (4.1%), tuberculous pleuritis; in one patient (0.7%), pancreatogenic pleuritis; and in one patient (0.7%), pulmonary embolism (PE) with pleuritis was diagnosed. Considering the small amount of tuberculous pleuritis, pancreatogenic pleuritis, and PE, these patients were not analyzed further.

In 44 (65.7%) of the 67 MPE patients, malignant cells were found in the pleural effusion.

**Figure 1:**
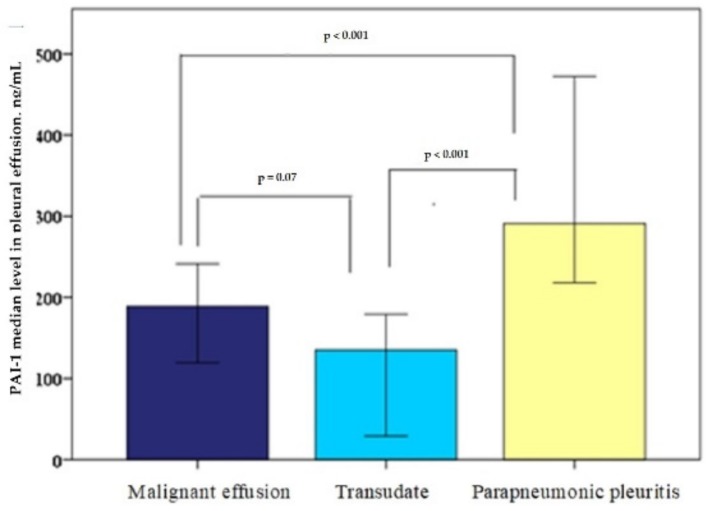
Median plasminogen activator inhibitor-1 (PAI-1) level in pleural effusion.

The median PAI-1 levels in the pleural effusion (ng/mL) were: for the cardiac hydrothorax group, 135 (20–236); for the malignant effusions group, 188 (73–287); for the parapneumonic pleuritis group, 291 (213–499). The PAI-1 level difference between the parapneumonic pleuritis and malignant pleural effusions was statistically reliable (*p* < 0.001); however, there was no statistically significant difference between the cardiac hydrothorax and malignant effusion PAI-1 values (*p* = 0.07). Additionally, the difference between the cardiac hydrothorax and the parapneumonic pleuritis was statistically reliable (*p* < 0.001), as shown in Figure 1.

**Figure 2:**
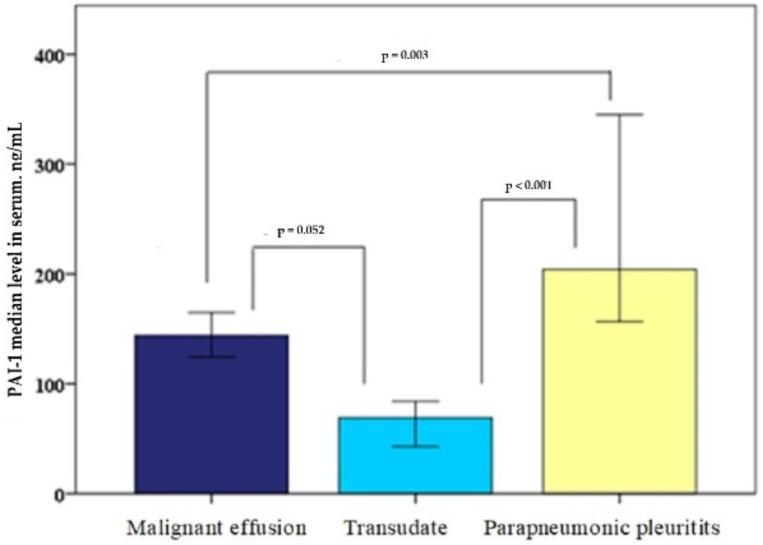
Plasminogen activator inhibitor-1 (PAI-1) median level in serum.

The PAI-1 (ng/mL) serum level in patients with MPE was 144 (77–207); in patients with cardiac hydrothorax, was 69 (34–166); and in patients with parapneumonic pleuritis, was 204 (151–412). Moreover, in serum, there was a statistically reliable difference between the parapneumonic pleuritis and MPE groups (*p* = 0.003) and the parapneumonic pleuritis and cardiac hydrothorax groups (<0.001); however, there was no statistically significant difference between the MPE and cardiac hydrothorax groups (*p* = 0.052), as shown in Figure 2.

The median PAI-1 effusion/serum ratios were as follows: for the cardiac hydrothorax group, 1.84 (0.18–6.74); for the malignant effusions group, 0.79 (0.38–3.14); for the parapneumonic pleuritis group, 1.05 (0.58–3.66). There was no statistically significant difference between all groups: MPE and cardiac hydrothorax: *p* = 0.82; cardiac hydrothorax and parapneumonic pleuritis: *p* = 0.11; and parapneumonic pleuritis and MPE: *p* = 0.43.

## 4. Discussion

The role of PAI-1 in pleural effusion formation seems to be controversial. Our study shows that the median PAI-1 level was the highest in the parapneumonic pleuritis group both in the effusion and the serum, resulting in a statistically significant difference from the cardiac hydrothorax and MPE groups. However, there was no statistically significant difference between PAI-1 levels in the pleural effusion and serum in cardiac hydrothorax and MPE groups.

There is little research about the role of PAI-1 in pleural effusion. In 1995, in 10 patients with empyema, in 9 patients with tuberculous pleuritis, in 31 patients with MPE, and in 3 patients with pleural effusion of an unclear etiology, PAI-1 and D-dimers were measured in plasma and in pleural effusion. It was established that the levels of D-dimers and PAI-1 were higher in pleural effusion than in plasma. In patients with tuberculous pleuritis and empyema, the PAI-1 level was higher than in patients with cardiac hydrothorax or MPE [7]; this is fully in line with our data, which established that the PAI-1 level in parapneumonic pleuritis was statistically reliably higher than in malignant effusions and cardiac hydrothorax.

In a study with 19 tuberculous pleuritis patients, 29 MPE patients, and 30 parapneumonic pleuritis patients, PAI-1 was measured in pleural effusion along with other markers. Depending on the location of the pleural effusion, the patients were divided into two groups: loculated (42 patients) and free pleural effusion (36 patients). In the loculated pleural effusion group, the PAI-1 level was much higher (114.9 vs. 94.1 pg/mL; *p* = 0.019). Obviously, the elevated PAI-1 points to reduced fibrinolyses in loculated effusions [8]. In another study, 64 patients with parapneumonic pleuritis were divided into two groups: noncomplicated (26 patients) and complicated loculated (38 patients) parapneumonic pleuritis. For noncomplicated parapneumonic pleuritis, the PAI-1 level was 43 pg/mL, but for the complicated cases, it was 104 pg/mL (*p* < 0.01), which points to reduced fibrinolytic activity in the pleural cavity during the formation of fibrin septa [9]. In a study with 30 parapneumonic pleuritis patients, the PAI-1 levels in pleural effusion were significantly higher in the Gram-positive culture group (160 ng/mL) and Gram-negative culture group (117 ng/mL) than in the uncomplicated culture negative group (58.0 ng/mL) [10]. In our case, the patients were not grouped depending on effusion loculation in the pleural cavity or Gram stain; however, in patients with parapneumonic pleuritis, the median PAI-1 level was higher (291 ng/mL, *p* < 0.001, indicating a statistically reliable result).

In general, the formation of fibrin is characteristic of parapneumonic pleuritis, and there is a tendency for the effusion to encapsulate, localizing the process. Studies have shown that inflammatory effusions do have an elevated PAI-1 level that facilitates the inhibition of fibrinolyses and the formation of fibrin [11,12]. This means that an increased formation of PAI-1 can be affected by the inflammation process. Our study also demonstrated that in parapneumonic pleuritis, the PAI-1 level is significantly higher than in malignant effusions.

Malignant pleural effusions do not tend to loculate, which means that no or very little fibrin forms in them that can be related to average PAI-1 activity [12,13]. Our data points to a significantly lower PAI-1 level in malignant pleural effusions in comparison to parapneumonic pleuritis (188 ng/mL and 291 ng/mL, *p* < 0.001). We assume that the average PAI-1 level in MPE does not promote the formation of fibrin and effusion loculation like with parapneumonic pleuritis, but does facilitate the spread of the pathological process. There is still an open question about what inhibits PAI-1 formation in MPE and how this can be prevented. As palliative therapy for MPE is based on process encapsulation with iatrogenic fibrin precipitation (pleurodesis), the answer to this question may improve MPE treatment strategies.

Our data show that the serum PAI-1 level was statistically reliably higher in parapneumonic pleuritis than in MPE and cardiac hydrothorax (204 ng/mL, *p* = 0.003 and < 0.001, respectively), which points to the systemic effect of this fibrin inhibitor.

A limitation of this study is the small number of patients used to analyze PAI-1 in different subgroups of MPE (different primary cancer localization, loculated versus free effusion, monomorphonuclear cell count).

## 5. Conclusions

The PAI-1 level in MPE and cardiac hydrothorax was statistically significantly lower than in parapneumonic pleuritis, which was a statistically reliable result. This might point to the fact that the fibrinolytic system’s activity is higher and that the process tends to spread during malignant diseases. Therefore, further studies of the fibrinolytic activity in MPE would be useful.
